# Molecular surveillance of resistance mutations in invasive populations of *Spodoptera frugiperda* in Europe, for evidence‐based pest control

**DOI:** 10.1002/ps.8849

**Published:** 2025-04-25

**Authors:** Konstantinos Mavridis, Vasiliki Evangelou, Alexandra M Grigoriadou, Dimitrios P Papachristos, John Vontas

**Affiliations:** ^1^ Institute of Molecular Biology and Biotechnology of the Foundation for Research and Technology Hellas (IMBB‐FORTH) Heraklion Greece; ^2^ Laboratory of Agricultural Entomology, Scientific Directorate of Entomology and Agricultural Zoology Benaki Phytopathological Institute Attica Greece; ^3^ Department of Crop Science Agricultural University of Athens Athens Greece

**Keywords:** *Spodoptera frugiperda*, fall armyworm, insecticide resistance, molecular diagnostics, integrated pest management

## Abstract

**BACKGROUND:**

The invasive fall armyworm (*Spodoptera frugiperda*, FAW), a highly destructive pest affecting more than 350 plant species, has recently invaded Europe raising urgent management concerns. Insecticide resistance profiling is essential to support evidence‐based pest control strategies. In this study, we analyzed target‐site insecticide resistance mutations in FAW populations from Greece to inform pest control strategies. In addition, DNA barcoding through cytochrome oxidase subunit 1 (*COI*) gene sequencing was used to trace the pest's geographic origin and potential invasion pathways.

**RESULTS:**

All *Spodoptera frugiperda* specimens in Greece were identified as the rice strain, exhibiting two almost balanced haplotypes (Haplotype 1: 58.6%; Haplotype 2: 41.4%), suggesting a likely origin from a single, genetically diverse source population. Resistance‐associated mutations were identified in the *ABCC2* gene (A > G single‐nucleotide polymorphism (SNP); up to 80.9%) and the *Ace‐1* gene (F290V: up to 37.5%; A201S: up to 3.85%), conferring resistance to *Bacillus thuringiensis* (Bt) and organophosphates/carbamates, respectively. By contrast, no resistance‐associated mutations were detected for other key insecticides (diamides, pyrethroids, oxadiazines, spinosyns, and avermectins), suggesting their current efficacy in Greece.

**CONCLUSION:**

This study provides a critical baseline for monitoring insecticide resistance in invasive FAW populations in Europe, supporting the development of sustainable integrated pest management strategies in line with the European Union Green Deal. Continuous monitoring with molecular diagnostics, alongside complementary bioassays, is recommended to mitigate the impact of FAW on European agriculture. © 2025 The Author(s). *Pest Management Science* published by John Wiley & Sons Ltd on behalf of Society of Chemical Industry.

## Graphical abstract

All *Spodoptera frugiperda* were rice strain with two haplotypes, likely from a common mixed source. *ABCC2* and *Ace‐1* mutations, conferring resistance to *Bacillus thuringiensis* and organophosphates/carbamates, respectively, were detected.

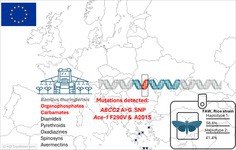

## 1 INTRODUCTION

*Spodoptera frugiperda* (J.E. Smith) (Lepidoptera: Noctuidae), commonly known as the fall armyworm (FAW), is a highly invasive pest originally native to the tropical and subtropical regions of the Americas. Since its first detection outside its native range in Western Africa in 2016, FAW has rapidly spread to more than 70 countries across Africa, Asia, Oceania, and Europe.^1^, ^2^ This expansion is facilitated by its strong flight capabilities, which enable long‐distance migration, and its adaptability to various climatic conditions, allowing it to thrive in diverse environments and continue its invasion across new territories.^2^

FAW primarily targets maize, its preferred host, but is capable of infesting more than 350 plant species, including key crops such as rice, sorghum, millet, and sugarcane.^2^, ^3^ In newly invaded regions like Australia and Europe, this pest poses a significant threat to food security because of its capacity to damage a wide range of economically important crops. As a result, these regions face urgent challenges in managing FAW populations to prevent large‐scale agricultural losses, necessitating immediate and effective pest control measures.^1^, ^3^

*S. frugiperda* populations are classified into two strains: the corn strain (C‐strain) and the rice strain (R‐strain), each exhibiting distinct invasion patterns and crop preferences. The corn strain primarily targets temperate regions and infests crops like maize, whereas the rice strain favors tropical climates, often affecting rice and pasture grasses. Both strains show high adaptability and can coexist sympatrically, leading to complex management challenges because of overlapping and prolonged infestations across multiple regions.^4^, ^5^, ^6^, ^7^, ^8^ Strain identification and invasion monitoring rely only on molecular methods, such as next generation sequencing, real‐time polymerase chain reaction (PCR), and DNA barcoding, which is based on mitochondrial cytochrome oxidase subunit 1 (*COI*) gene sequencing.^9^, ^10^, ^11^

In Europe, *S. frugiperda* is regulated as a quarantine pest for the European Union (EU) according to Commission implementing regulation (EU) 2019/2072 and is nominated as a priority pest in the Commission delegated regulation (EU) 2019/1702.^12^ FAW has recently been recorded in several regions across Europe, signaling its spread throughout the continent. The pest was first reported in Türkiye in 2022,^13^ followed by detections in Cyprus in January 2023.^14^ Further records include its presence in Madeira, Portugal,^15^ and Malta,^16^ both in September 2023, and more recently in Romania in November 2023.^17^ In Greece, *S. frugiperda* was firstly detected in September 2023 in various regions across the country.^18^ Currently, the pest is classified as ‘present, under eradication,’ with intensified surveys and eradication measures being implemented.^18^, ^19^ These records indicate that *S. frugiperda* is establishing itself in several southern European countries, underscoring the need for continuous monitoring and proactive management efforts to control its spread. In particular, Greece's favorable climate, availability of host crops and the current detections raise concerns about the potential for the pest's permanent establishment, making proactive management measures crucial.^2^, ^20^

Chemical insecticides are among the most used strategies for managing FAW's populations, but FAW has demonstrated an alarming capacity to adapt and develop resistance to a broad spectrum of insecticides, including *Bacillus thuringiensis* (Bt) toxins, diamides, spinosyns, avermectins, organophosphates, and pyrethroids, each targeting different biological pathways in the pest, posing significant challenges for effective control.^3^ Molecular studies have documented various mechanisms behind this resistance, including metabolic resistance,^21^, ^22^, ^23^ and mutations in key target genes (target‐site mutations).^24^, ^25^

Bt transgenic crops expressing *Cry* toxins have revolutionized pest management by offering highly specific control of lepidopteran pests, including FAW. However, resistance to Bt crops has emerged as a major challenge because of the pest's rapid adaptation. The primary mechanism of resistance to Bt toxins in the FAW is linked to mutations in the ATP‐binding cassette subfamily C2 (*ABCC2*) transporter gene, such as the +GC insertion (R1 resistance allele), the A > G single‐nucleotide polymorphism (SNP) (R2 resistance allele),^26^ the GY deletion and P799K/R substitutions.^27^, ^28^ These genetic alterations disrupt Bt toxin receptors in the insect's midgut, resulting in substantial resistance levels. Similarly, newer insecticides like diamides, which target the ryanodine receptor, have also seen early signs of resistance because of mutations in the *RyR* gene (I4790M/K and G4946E), although resistance levels remain lower compared with other classes.^29^, ^30^ Among these, the G4946E mutation is a well‐characterized resistance marker in other lepidopteran species (e.g., *Plutella xylostella* ^31^), but it has not yet been identified in *S. frugiperda*. Spinosyns, which affect the nicotinic acetylcholine receptor (nAChR), also face resistance challenges. Specific mutations such as G275E and the IIA deletion in the *nAChR* gene have been identified, reducing sensitivity to these insecticides^32^ and could complicate FAW control efforts. Their presence in *S. frugiperda*, however, has yet to be confirmed. The resistance mechanism for avermectins involves the glutamate‐gated chloride channel (*GluCl*) gene. Research has demonstrated that knockdown of *GluCl* leads to decreased susceptibility to emamectin benzoate^33^ and field studies in China have confirmed the presence of A308V and G314D mutations in the *GluCl* gene of *S. frugiperda*.^34^ Resistance to organophosphates (OPs) and carbamates (CBs) in *S. frugiperda* is primarily associated with mutations in the *Ace‐1* gene (A201S, G227A, F290V), which reduce the effectiveness of acetylcholinesterase inhibitors.^24^, ^35^ Similarly, resistance to pyrethroids and oxadiazines has been linked to mutations in the voltage‐gated sodium channel (*vgsc*) gene, including M918T, T929I, L932F, I936V, L1014F, F1020S^36^ for pyrethroids and F1845Y and V1848I^37^, ^38^ for oxadiazines. However, unlike pyrethroid‐associated mutations, there is no evidence of oxadiazine‐related mutations occurring in *S. frugiperda*.

Increasing evidence suggests that the overexpression of detoxification genes, such as cytochrome P450 monooxygenases (CYPs), glutathione *S*‐transferases, and carboxylesterases (CCEs), is linked to metabolic resistance in *S. frugiperda*.^21^, ^22^, ^23^ Functional validation has been demonstrated in specific cases, including the *CYP9A* subfamily, which is associated with resistance to insecticides like indoxacarb, abamectin, and the pyrethroid esfenvalerate.^39^

Alarmingly, cases of insecticide resistance in *S. frugiperda* have surged rapidly over the past seven years. In 2017, this pest was resistant to at least 29 insecticidal active ingredients.^3^ Since then, resistance to 47 active ingredients in different chemical classes have been reported in the Arthropod Pesticide Resistance Database (APRD) and 263 cases of insecticide resistance in *S. frugiperda* globally (APRD 2024; https://www.pesticideresistance.org/). Building on this alarming trend, the ability of FAW to develop resistance to multiple classes of insecticides, including, for example, Bt toxins, pyrethroids, and spinosyns, highlights the need for integrated pest management (IPM) approaches^29^, ^40^, ^41^ to reduce reliance on single insecticide classes. For example, a single *S. frugiperda* population in Puerto Rico was found to have resistance against flubendiamide, chlorantraniliprole, methomyl, thiodicarb, permethrin, chlorpyriphos, zeta‐cypermethrin, deltamethrin, triflumuron and spinetoram.^42^

Here, we monitored the presence and frequency of target‐site insecticide resistance mutations of invasive *S. frugiperda* populations in Greece, to provide evidence for precise pest control management applications. Haplotype profiles occurring in the different regions after invasion were also analyzed, aiming to trace the geographical origin of the pest population and potential points of invasion.

## 2 MATERIALS AND METHODS

### 2.1 *Spodoptera frugiperda* populations and DNA preparation

**Figure 1:**
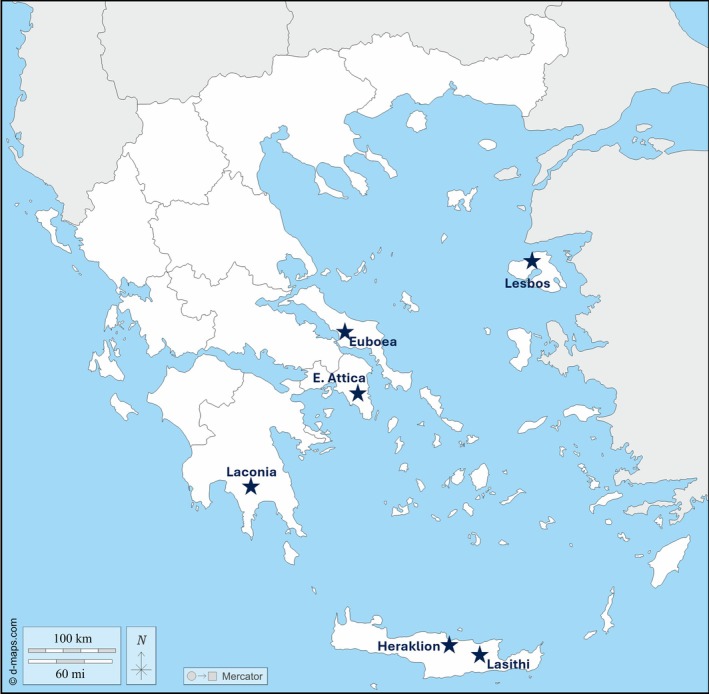
Map of Greece displaying the collection sites of *Spodoptera frugiperda* populations, marked with dark blue stars. The identified sites include East Attica, Euboea, Laconia, Lesbos, Heraklion, and Lasithi, indicating key regions where the species has been detected and subjected to molecular monitoring in this study. The base layers of the map were obtained from d‐maps.com ^43^ (https://d‐maps.com/carte.php?num_car=5706&lang=en accessed 21 October 2024).

Samples were collected as previously described,^18^ within the framework of Greece's official national survey program for quarantine pests, coordinated by the National Plant Protection Organization since 2009. Pheromone traps targeting male *S. frugiperda* were placed primarily in maize, rice, and Solanaceae fields across the country. In 2023, following the first detections of *S. frugiperda*, the trap network was expanded, and additional inspections were conducted in affected regions. Six study populations (Lesbos, Euboea, East Attica, Laconia, Heraklion, and Lasithi) were sampled during this intensified survey effort (Fig. 1; Supporting Information, Table S1).

The insects were identified morphologically based on keys and molecularly by applying species specific real‐time PCR, as described in the European and Mediterranean Plant Protection Organization (EPPO) diagnostic protocol.^18^ Total genomic DNA, belonging to 111 males, was extracted from their thorax by using the DNeasy Blood and Tissue Kit (Qiagen, Germantown, MD, USA), following the manufacturer's protocol. The extracted DNA was suspended in 50 μL of the kit's elution buffer (AE) and stored at −20 °C until further analysis.

**Table 1:** Species ID and haplotype frequencies based on barcode sequences of *Spodoptera frugiperda* populations in the different invasion sites

Population	Species, strain	Haplotype 1	Haplotype 2
East Attica	*S. frugiperda*, rice strain		
Frequency (%)	100	50.0	50.0
No. individuals (*n*)	20	10	10
Euboea	*S. frugiperda*, rice strain		
Frequency (%)	100	65.0	35.0
No. individuals (*n*)	20	13	7
Laconia	*S. frugiperda*, rice strain		
Frequency (%)	100	50.0	50.0
No. individuals (*n*)	20	10	10
Lesbos	*S. frugiperda*, rice strain		
Frequency (%)	100	61.9	38.1
No. individuals (*n*)	21	13	8
Heraklion	*S. frugiperda*, rice strain		
Frequency (%)	100	46.2	53.8
No. individuals (*n*)	13	6	7
Lasithi	*S. frugiperda*, rice strain		
Frequency (%)	100	76.5*	23.5*
No. individuals (*n*)	17	13	4
Total	*S. frugiperda*, rice strain		
Frequency (%)	100	58.6	41.4
No. individuals (*n*)	111	65	46

* *P* = 0.049, statistically significant difference between haplotypes; no other statistically significant difference was observed.

### 2.2 Strain and haplotype identification using *COI* gene sequencing

A molecular analysis was conducted to determine the species, following the standard sequencing protocol using the primers LCO‐1490 (5′‐GGTCAACAAATCATAAAGATATTGG‐3′) and HCO‐2198 (5′‐TAAACTTCAGGGTGACCAAAAAATCA‐3′), as described by EPPO.^44^ PCR analysis was performed using thermostable hot‐start *Taq* DNA polymerase (Platinum; Invitrogen, Carlsbad, CA, USA), generating 50 μL of PCR product, which was verified via electrophoresis on a 1.2% (w/v) agarose gel. If the expected size of approximately 700 bp was confirmed, the remaining 45 μL of the PCR product was purified using the NucleoFast 96 PCR Clean‐up Kit (Macherey‐Nagel, Dueren, Germany) following the manufacturer's instructions. Sanger sequencing was subsequently performed in‐house using the SeqStudio Genetic Analyzer System (Applied Biosystems, Waltham, MA, USA).

The sequencing data obtained were processed, optimized, and aligned using Geneious Prime 2023.0.1 software (https://www.geneious.com/). The resulting consensus sequences were 658 bp in length, excluding primers, and contained no gaps or missing nucleotides. Species identity was verified by comparing these sequences against *S. frugiperda* entries in three molecular databases: the National Center for Biotechnology Information (NCBI; https://blast.ncbi.nlm.nih.gov/), the Barcode of Life Database (BOLD; https://www.boldsystems.org/), and the EPPO quarantine‐bank (EPPO‐Q‐bank; https://qbank.eppo.int/blast?db=arthropods). The 111 sequences generated from the collected samples were analyzed for variable sites and haplotype diversity using DnaSP 6.0 software. The identified haplotypes were further compared with sequences deposited in the NCBI and BOLD databases to investigate their occurrence and potential geographic origin. To investigate the potential origin of the invasive populations, each haplotype sequence was compared against the top 100 matching entries in the BOLD database (100% similarity), using the platform's built‐in neighbor‐joining tree construction tool. In addition, the sequencing results were used to determine the strain identity (rice or corn) of the sampled individuals by comparing them with established reference sequences: Sf_corn_AY714298.1 for the corn strain and Sf_rice_U72978.1 for the rice strain.

### 2.3 Detection of insecticide resistance‐associated mutations using quantitative PCR

Genomic DNA extracted from 111 individual *S. frugiperda* insects collected across Greece were used in TaqMan diagnostic assays to analyze resistance markers. Specifically, each sample was tested for: (i) the +GC insertion and A > G substitution in the *ABCC2* gene, associated with resistance to Bt and its insecticidal proteins [Insecticide Resistance Action Committee (IRAC) mechanism 11], using the assay of Flagel *et al*.^26^; and (ii) the I4790M and I4790K target‐site mutations in the *RyR* gene, associated with resistance to ryanodine receptor modulators (IRAC mechanism 28), using the assay described in Okuma *et al*.^30^ Primer and probe sequences are provided in Table S2. PCR reactions were performed on a CFX Opus Real‐time PCR System (Bio‐Rad, Hercules, CA, USA) with the following thermal protocol: an initial incubation at 98 °C for 3 min, followed by 45 amplification cycles at 95°C for 5 s and 60 °C for 20 s. Each reaction included 5.0 μL of 2× KAPA Probe Force qPCR Master Mix (Kapa Biosystems, Wilmington, MA, USA), 500 nm primers, 300 nm probes, and 1.0 μL of genomic DNA, adjusted to a final volume of 10 μL with diethylpyrocarbonate (DEPC)‐treated water. Genotyping was conducted using the Allelic Discrimination module in Bio‐Rad CFX MAESTRO 2.3 software (version 5.3).

### 2.4 Detection of insecticide resistance‐associated mutations using Sanger sequencing

The following resistance‐associated mutations in *S. frugiperda* were monitored using PCR amplification and sequencing of the relevant gene regions: (i) A201S, G227A, and F290V (*Ace‐1* gene); (ii) G275E and IIA deletion (*nAChR*); (iii) M918T, T929I, L932F, I936V, L1014F, F1020S, F1845Y, and V1848I (*vgsc*); and (iv) A308V and G314D (*GluCl*). Established assays, as described by Wang *et al*.,^36^ were used for these mutations. For additional mutations, *de novo* primers were designed to amplify and sequence specific gene regions: (i) GY deletion and P799K/R (*ABCC2*), using a cycling protocol of 95 °C for 3 min, followed by 40 cycles of 95 °C for 30 s, 52 °C for 30 s, and 72 °C for 1 min, with a final elongation at 72 °C for 5 min; and (ii) G4946E (*RyR*), with a cycling protocol of 95 °C for 5 min, followed by 40 cycles of 95 °C for 30 s, 56 °C for 45 s, and 72 °C for 45 s, finishing with a final elongation at 72 °C for 10 min. All PCR reactions were performed using the KAPA Taq PCR Kit (Kapa Biosystems) with 1.0 μL genomic DNA as a template. PCR fragments were visualized on a 1.5% (w/v) agarose gel, purified using the Nucleospin PCR & Gel Clean‐Up Kit (Macherey‐Nagel), and sequenced by Sanger sequencing (GENEWIZ, Azenta Life Sciences, Leipzig, Germany) using the forward primer. Sequences were analyzed with the BioEdit Sequence Alignment Editor version 7.2 (https://bioedit.software.informer.com/7.2/). Primer sequences along with their corresponding reference GenBank accession numbers are provided in Table S2.

### 2.5 Data analysis

The statistical significance of differences between haplotype frequencies was assessed using the non‐parametric binomial test in SPSS (v17.0). Mutation frequencies were calculated using the mutant allele frequency (MAF) formula, which accounts for both heterozygous (het) and homozygous mutant (hom) individuals. For each mutation, the percentage MAF (%MAF) was calculated using the equation: $\%\mathrm{MAF}=\left(\frac{\left(2\times \mathrm{hom}+\mathrm{het}\right)\;}{2n}\right)\times 100$, where *n* is the total number of insects analyzed per population.

## 3 RESULTS

### 3.1 Strain identification and haplotype frequencies of *Spodoptera frugiperda* populations

All sampled individuals were confirmed as *S. frugiperda* based on submitted sequences, achieving 100% similarity. In addition, all individuals were identified as the rice strain, with 100% of each population belonging to this strain (Table 1; Supporting Information, Fig. S1).

The haplotype frequencies of *S. frugiperda* populations from six regions in Greece were analyzed based on *COI* sequences. Two distinct haplotypes were observed across the populations, reflecting mixed populations with no distinct separation between haplotypes (Haplotype 1 = 58.6%; Haplotype 2 = 41.4%). More precisely, in East Attica and Laconia a total of 20 individuals were sampled, with an equal distribution of the two haplotypes (50% for both). In Euboea, of 20 individuals, 65% belonged to Haplotype 1 and 35% were assigned to Haplotype 2. In Lesvos, among 21 individuals, Haplotype 1 comprised 61.9% of the population and Haplotype 2 accounted for 38.1%. In the Heraklion population, consisting of 13 individuals, Haplotype 1 was present in 46.2%, and Haplotype 2 in 53.8%. None of these differences were statistically significant. However, in Lasithi, among 17 individuals, a statistically significant difference (*P* = 0.049) was observed between the haplotypes, with 76.5% belonging to Haplotype 1 and 23.5% to Haplotype 2 (Table 1). Haplotype 1 is genetically closest, in descending order, to sequences from South Africa, Ecuador, Australia, Canada, USA, Mexico, and Brazil (Supporting Information, Fig. S2). Haplotype 2 is genetically closest to sequences from the following regions: South Africa, Canada, Puerto Rico, Réunion (east of Madagascar), Australia, Argentina, USA, Dominican Republic, Brazil, and French Guiana (Supporting Information, Fig. S3).

### 3.2 Molecular profiling of mutations associated with insecticide resistance

The resistance of *S. frugiperda* populations to Bt insecticidal proteins, targeting the ATP‐binding cassette transporter gene (*ABCC2*) product, was analyzed in six invasion sites across Greece. Four specific mutations were examined: R1 (+GC insertion), R2 (A > G SNP), GY deletion, and P799K/R.

The R2 (A > G) resistant mutation in the *ABCC2* gene was prevalent with MAF values varying from 70.0% to 80.9% (frequencies of homozygous mutant individuals varying from 58.8% to 71.4%). The highest frequency was observed in the Lesvos population (80.9%), and the lowest was in Euboea and Laconia, both at 70.0%. Analysis indicated the presence of both heterozygous and homozygous resistant individuals in all populations. The highest proportion of homozygous mutant individuals was found in Lesvos (71.4%) and the lowest proportion in Lasithi (58.8%).

**Figure 2:**
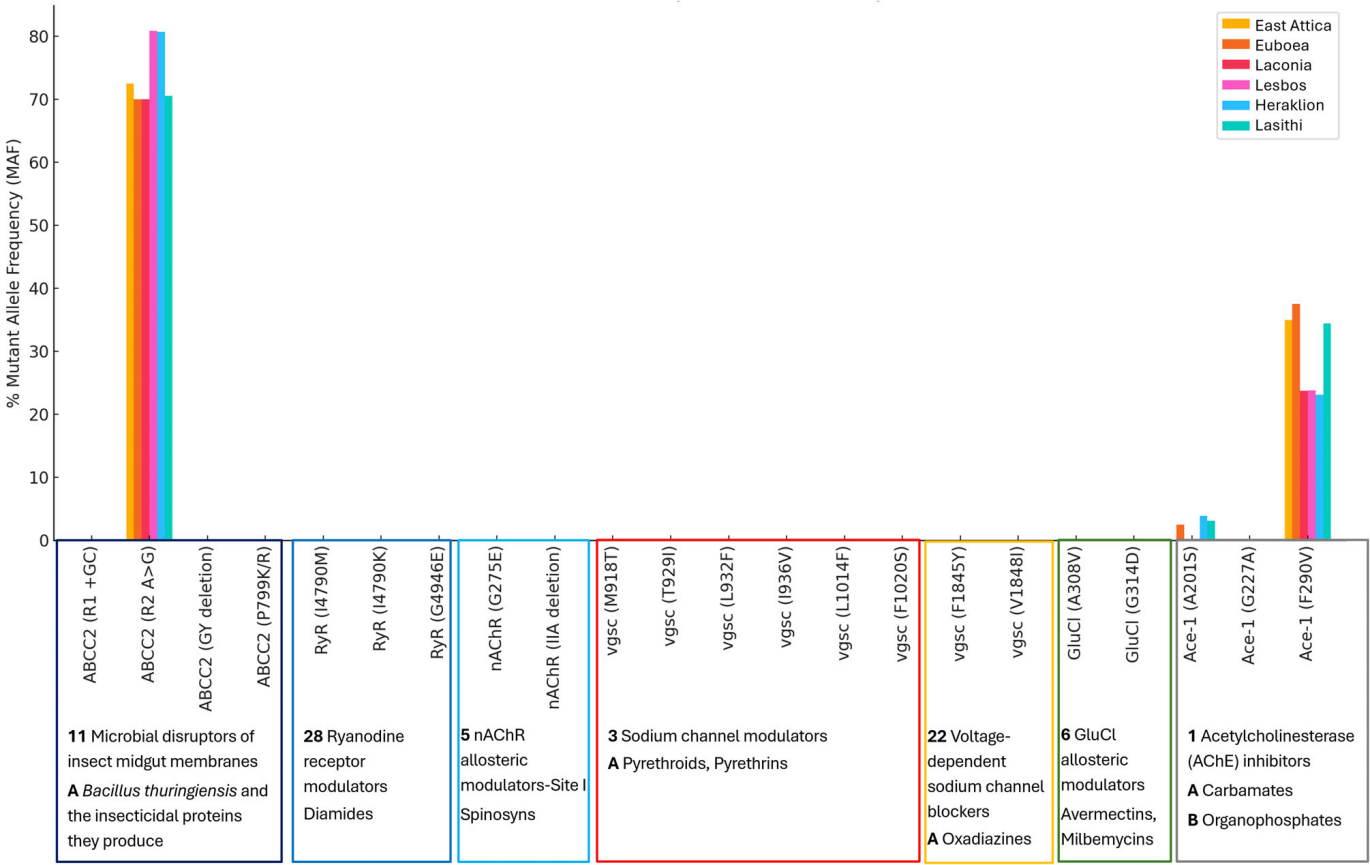
Resistant mutation allele frequencies (%) across various populations. Mutations are grouped by their respective mechanisms.

No resistant alleles were detected in any of the populations across the six sites for R1 (+GC), GY deletion, and P799K/R mutations (Table 2 and Fig. 2).

**Table 2:** Monitoring for mutations associated with Bt insecticidal proteins resistance (IRAC mechanism 11A)

IRAC mechanism	11A Microbial disruptors of insect midgut membranes			
	Bt and the insecticidal proteins they produce			
Gene	*ABCC2*	*ABCC2*	*ABCC2*	*ABCC2*
Mutation	R1: + GC	R2: A > G	GY deletion	P799K/R
Population				
East Attica				
Resistant mutation allele frequencies (%)	0.00	72.50	0.00	0.00
Het/Hom	0/0	5/12	0/0	0/0
No. of alleles (*N*)	40	40	36	36
Euboea				
Resistant mutation allele frequencies (%)	0.00	70.00	0.00	0.00
Het/Hom	0/0	4/12	0/0	0/0
No. of alleles (*N*)	40	40	30	30
Laconia				
Resistant mutation allele frequencies (%)	0.00	70.0	0.00	0.00
Het/Hom	0/0	4/12	0/0	0/0
No. of alleles (*N*)	40	40	34	34
Lesbos				
Resistant mutation allele frequencies (%)	0.00	80.9	0.00	0.00
Het/Hom	0/0	4/15	0/0	0/0
No. of alleles (*N*)	42	42	36	36
Heraklion				
Resistant mutation allele frequencies (%)	0.00	80.7	0.00	0.00
Het/Hom	0/0	3/9	0/0	0/0
No. of alleles (*N*)	26	26	24	24
Lasithi				
Resistant mutation allele frequencies (%)	0.00	70.6	0.0	0.0
Het/Hom	0/0	4/10	0/0	0/0
No. of alleles (*N*)	34	34	28	28

IRAC, Insecticide Resistance Action Committee; Het, number of heterozygous individuals; Hom, number of homozygous mutant individuals.

Mutations linked to resistance against acetylcholinesterase (AChE) inhibitors, specifically OPs and CBs, were monitored in the six *S. frugiperda* populations. Three mutations in the *Ace‐1* gene were analyzed: A201S, G227A, and F290V. Resistance‐related mutations were rare for A201S. Most populations showed 0.00% allele frequencies, except for Euboea (2.5%), Heraklion (3.85%), and Lasithi (3.13%), where resistant alleles were present in a small proportion of individuals in a heterozygous form. No resistant alleles were detected for the G227A mutation across all populations, with a consistent 0.00% frequency for this mutation in every region. The F290V mutation showed varying levels of resistance across the populations. The highest frequencies were observed in Euboea (37.5%) and East Attica (35.0%), where both heterozygous and homozygous resistant individuals were present. Other populations, such as Lasithi (34.4%), Laconia (23.7%), Lesvos (23.8%), and Heraklion (23.1%), also showed moderate frequencies of this mutation, primarily in heterozygous individuals (Table 3 and Fig. 2).

**Table 3:** Monitoring for mutations associated with organophosphate / carbamate resistance (IRAC mechanism 1AB)

	1 Acetylcholinesterase (AChE) inhibitors		
	A Carbamates		
IRAC mechanism	B Organophosphates		
Gene	*Ace‐1*	*Ace‐1*	*Ace‐1*
Mutation	A201S	G227A	F290V
Population			
East Attica			
Resistant mutation allele frequencies (%)	0.00	0.00	35.0
Het/Hom	0/0	0/0	10/2
No. of alleles (*N*)	40	40	40
Euboea			
Resistant mutation allele frequencies (%)	2.50	0.00	37.5
Het/Hom	1/0	0/0	13/1
No. of alleles (*N*)	40	40	40
Laconia			
Resistant mutation allele frequencies (%)	0.00	0.00	23.7
Het/Hom	0/0	0/0	7/1
No. of alleles (*N*)	38	38	38
Lesbos			
Resistant mutation allele frequencies (%)	0.00	0.00	23.8
Het/Hom	0/0	0/0	6/2
No. of alleles (*N*)	42	42	42
Heraklion			
Resistant mutation allele frequencies (%)	3.85	0.00	23.1
Het/Hom	1/0	0/0	6/0
No. of alleles (*N*)	26	26	26
Lasithi			
Resistant mutation allele frequencies (%)	3.13	0.00	34.4
Het/Hom	1/0	0/0	7/2
No. of alleles (*N*)	32	32	32

Het, number of heterozygous individuals; Hom, number of homozygous mutant individuals.

The resistance of *S. frugiperda* populations to diamides, which target the ryanodine receptor (RyR), was also assessed in the six invasion sites. Specifically, three mutations known to confer resistance (I4790M, I4790K, and G4946E) were investigated. No resistant alleles for any of the three mutations were detected (Fig. 2 and Supporting Information, Table S3).

Mutations linked to resistance against spinosyns were monitored in six *S. frugiperda* populations. Two resistance‐associated mutations, G275E and IIA deletion in the *nAChR* gene, were analyzed, but the analysis indicated that *S. frugiperda* populations in Greece do not currently carry mutations associated with resistance to nAChR channel blockers (Fig. 2; Supporting Information, Table S4).

The resistance of *S. frugiperda* populations to pyrethroids, which target sodium channel modulators, was investigated across the six invasion sites. Six specific mutations in the *vgsc* gene were analyzed: M918T, T929I, L932F, I936V, L1014F, and F1020S. The resistant mutation allele frequencies were recorded for each population. No resistant alleles were detected for any of the six mutations in any of the populations studied (Fig. 2; Supporting Information, Table S5).

The potential for resistance to oxadiazines, a class of insecticides targeting voltage‐dependent sodium channel blockers, was assessed in *S. frugiperda* populations across six Greek invasion sites. Two specific mutations in the *vgsc* gene were analyzed, F1845Y and V1848I, and no resistant alleles were detected for either mutation (Fig. 2; Supporting Information, Table S6).

The potential for resistance to avermectins, a class of insecticides acting as GluCl allosteric modulators, was assessed in *S. frugiperda* populations across six regions of Greece. Two specific mutations in the *GluCl* gene were analyzed: A308V and G314D. No resistant alleles were detected for either mutation in any of the populations sampled (Fig. 2; Supporting Information, Table S7).

## 4 DISCUSSION

Having arrived in Africa in 2016, in Western Asia in 2019 and in mainland EU in 2023, the polyphagous pest *S. frugiperda* constitutes a high risk to European agriculture.^1^, ^2^ Recognized as a priority pest in the EU since 2019 (2019/1702),^12^ it carries a high risk of establishment and spread, especially in the context of climate crisis^20^ As Europe experiences climate changes, including rising temperatures, reduced rainfall, and more frequent extreme weather events, regional variations in these conditions are expected to directly affect the fitness and distribution of *S. frugiperda*.^45^ These climate shifts will also influence pest dynamics indirectly, because they alter the distribution of host plants and natural enemies, further complicating management efforts.^2^, ^20^

Immediately following the detection of S. *frugiperda* in Greece, this study was implemented to perform a comprehensive molecular characterization of this invasion. The primary goals were to determine the strain and haplotype distributions and assess the insecticide resistance profile of the *S*. *frugiperda* populations that invaded Greece, and consequently Europe, thus establishing a foundational understanding of the pest's genetic diversity and resistance potential in the region. Such a baseline will provide essential information for monitoring future resistance trends and improving pest management strategies in Europe.

All sampled individuals were confirmed as *S. frugiperda* based on *COI* sequencing and were identified as belonging to the rice strain, which is renowned for its adaptability and aggressive infestation patterns.^4^, ^5^, ^8^ The rice strain has also been reported in Australia,^46^ Pakistan,^47^ South America,^48^ and various African countries.^49^, ^50^ Although our strain determination was based on *COI* mitochondrial markers, which are commonly used for this purpose, recent studies suggest that such markers may not reliably distinguish between the corn and rice strains. In fact, whole‐genome analyses indicate that invasive *S. frugiperda* populations globally—including those with *COI* profiles typically associated with the rice strain—are genomically closer to the corn strain, emphasizing the need for complementary nuclear markers in strain classification.^51^, ^52^ Haplotype analysis identified two distinct haplotypes among *S. frugiperda* populations, with Haplotype 1 accounting for 58.6% and Haplotype 2 for 41.4% of the 111 males analyzed. The two haplotypes were present across all sampled regions in almost balanced proportions, indicating a likely origin from a genetically mixed source population rather than from distinct, separate lineages. This distribution pattern implies that the Greek FAW populations could have originated from a single source with a heterogeneous genetic background, possibly spread through natural migration or human‐facilitated transport. A plausible origin for the *S. frugiperda* invasion in Europe and Greece could be from North Africa, as suggested by recent studies,^20^ or from nearby regions such as Türkiye.^13^

To provide insights for developing targeted management strategies, a comprehensive insecticide resistance profile (23 mutations linked to 7 insecticide resistance mechanisms) was compiled, across the 6 invasion sites.

Among four mutations linked to Bt resistance, only the R2 (A > G) mutation, leading to aberrant splicing and non‐functional ABCC2 receptor was detected, with the proportion of homozygous mutants, the only genotype showing a resistance phenotype, reaching as high as 71.4%, suggesting potential challenges in using Bt‐based control methods. The ABBC2‐R2 resistance mechanism has been previously reported in *S. frugiperda* populations from Puerto Rico and associated with phenotypic resistance.^26^ However, this mechanism has not been fully functionally validated and its exact contribution to the Bt resistance phenotype is unknown. The remaining mutations (R1: +GC insertion, GY deletion, and P799K/R), which have been functionally validated to confer strong resistance phenotypes against various Bt toxins,^28^, ^53^ were not detected.

Regarding OP and CB resistance, the A201S mutation in the *Ace‐1* gene was rare, found only in the heterozygous form in Euboea, Heraklion, and Lasith, with low frequencies. The G227A mutation was absent across all sites. The F290V mutation showed varying resistance levels, with high frequencies in Euboea (37.5%) and East Attica (35.0%), indicating possible resistance levels to acetylcholinesterase inhibitors in these areas. Similar patterns of *Ace‐1* mutations have been observed in *S. frugiperda* in Kenya and in other invasive lepidopteran pests like *Tuta absoluta* in Iran, indicating that resistance alleles may often accompany invasive populations. *Ace‐1* mutations have also been previously found in various countries globally, for example China,^38^ Brazil, Puerto Rico, Indonesia and Kenya.^24^ The qualitatively and quantitatively common resistance profile of the invading populations (Fig. 2) suggests that the invasion likely originated from a common population, rather than from separate, distinct lineages.

No resistance‐associated alleles were detected in Greek *S. frugiperda* populations for diamide (*RyR*: I4790M, I4790K, G4946E), spinosyn (*nAChR*: G275E, IIA deletion), pyrethroid (*vgsc*: M918T, T929I, L932F, I936V, L1014F, F1020S), oxadiazine (*vgsc*: F1845Y, V1848I), or avermectin (*GluCl*: A308V, G314D) insecticides, suggesting these classes may still be effective options. However, some of these resistance mechanisms have been documented in *S. frugiperda* populations globally, such as *RyR* and *vgsc* mutations in Brazil and China,^24^, ^30^, ^36^, ^38^ and *GluCl* mutations in China.^34^ Such resistance mechanisms could emerge in Greece and other invaded EU countries under selective pressure, particularly if these genotypes are introduced via new invasion events or currently exist at undetectable frequencies.

The identification of mutations in the *ABCC2* and *Ace‐1* genes, associated with resistance to Bt toxins and OPs/CBs respectively, reinforces the need for IPM approaches and proactive molecular monitoring to support sustainable *S. frugiperda* management in Europe. Resistance management strategies should prioritize the use of insecticides with different modes of action, while integrating molecular diagnostics to detect resistance early and guide evidence‐based interventions.

In Greece, although *S. frugiperda* is a newly introduced pest, insecticide products already approved for *Spodoptera* spp. (e.g., *Spodoptera littoralis*, *Spodoptera exigua*) are currently authorized for its control in all relevant crops. Active substances, approved under the national emergency response framework in line with EU regulations (EU 2016/2031, EU 2023/1134), include pyrethroids (lambda‐cyhalothrin, deltamethrin), diamides (chlorantraniliprole), avermectins (emamectin benzoate, abamectin), botanical insecticides (azadirachtin A), ecdysone agonists (tebufenozide), and microbial Bt products (Bt *kurstaki* strains 2348 and 351). By contrast, in the EU transgenic Bt maize cultivation is limited to a single approved event (MON810), primarily grown in Spain and Portugal,^54^ whereas OPs/CBs are not used against lepidopteran pests in Greece. Notably, compounds such as diamides and emamectin benzoate have demonstrated strong efficacy against several lepidopteran pests^55^, ^56^, ^57^, ^58^ and may serve as valuable tools in *S. frugiperda* resistance management strategies in Greece and Europe. Supported by our findings that show an absence of relevant resistance mechanisms, these insecticides could be prioritized in the short term, although continuous resistance monitoring remains essential to detect emerging resistance traits and ensure sustainable long‐term management.

Although our study provides valuable insights into resistance profiling, it has some limitations. Metabolic resistance involving validated detoxification enzymes (e.g., *CYP9A* subfamily^39^) in *S. frugiperda* was not assessed, and certain monitored markers (e.g., the *ABCC2* R2: A > G mutation^26^) are not fully characterized in terms of resistance phenotype intensity, spectrum, and fitness cost. In addition, comprehensive bioassays would be beneficial to identify any potential novel or uncharacterized resistance mechanisms.

A particularly important advancement would be the development of a simple, in‐field molecular diagnostic tool for detecting insecticide resistance in *S. frugiperda*. Such a tool would provide critical support for evidence‐based pest control, enabling rapid, targeted interventions in alignment IPM principles. By quickly identifying resistance markers, these tools allow real‐time tracking of resistance mechanisms, guiding the choice of effective treatments and minimizing over‐reliance on limited insecticide options—a strategy proven to extend the effectiveness of control measures.^59^, ^60^ When the invasive tomato pest *Tuta absoluta* spread across Europe, the lack of such diagnostic tools led to widespread, indiscriminate pesticide use as farmers resorted to any available chemical option, often with low efficacy and environmental costs caused by repetitive, high‐dosage applications.^61^, ^62^ Implementing diagnostic tools early in an invasion could help avoid similar situations, supporting timely, evidence‐based control strategies to manage resistance and safeguard crop production more sustainably.

## 5 CONCLUSION

This study provides a comprehensive baseline for insecticide resistance in invasive *S. frugiperda* populations in Greece and Europe, offering a reference point for monitoring future resistance development. This approach aligns with IPM principles and the EU Green Deal's commitment to sustainable agriculture by reducing pesticide use and advancing alternative methods. Effective management of *S. frugiperda* in Europe relies on biological controls (e.g., beneficial predators, parasitoids, entomopathogenic nematodes), alternative strategies (e.g., pheromones for mating disruption, low‐toxicity pesticides), and, crucially, continuous resistance monitoring to adapt evidence‐based IPM strategies promptly and maintain long‐term control efficacy.^2^ This approach is particularly critical when managing a multi‐resistant pest like *S. frugiperda* because it may spread rapidly into new territories, helping to mitigate resistance risks and maintain control efficacy over time.^41^

## CONFLICT OF INTEREST

The authors declare that there is no conflict of interest.

## Supporting information


**Data S1:** Supporting Information.

## Data Availability

The data that support the findings of this study are available from the corresponding author upon reasonable request.
