# Supplementary material for: Molecular surveillance of resistance mutations in invasive populations of Spodoptera frugiperda in Europe, for evidence‐based pest control
Source: Pest Manag Sci. 2025 Apr 25;81(8):4821–30. doi: 10.1002/ps.8849 (PMC12268801; doi:10.1002/ps.8849)
Supplement: Supplementary file 1 — Data S1: Supporting Information. [file PS-81-4821-s001.docx]

**SUPPLEMENTAL MATERIAL**

**Supplemental Table S1** Detailed characteristics of the study’s sampling sites.

| **Site (Unit Region)** | **Decimal degrees (DD)** | | **N of insects** | **Collection Date** |
| --- | --- | --- | --- | --- |
|  | **Latitude** | **Longitude** |  |  |
| East Attica | 37.928568759397 | 23.908364437196 | 20 | 14/09/2023 |
| Euboea | 38.436390930275 | 23.675048162174 | 3 | 22/09/2023 |
|  | 38.481960257042 | 23.633672134781 | 3 | 08/11/2023 |
|  | 38.451618735342 | 23.674074508966 | 3 | 08/11/2023 |
|  | 38.41511375283 | 23.64718272446 | 2 | 08/11/2023 |
|  |  |  | 1 | 21/11/2023 |
|  | 38.457730784808 | 23.646723110542 | 1 | 08/11/2023 |
|  |  |  | 1 | 21/11/2023 |
|  | 38.450186864937 | 23.658621171883 | 2 | 08/11/2023 |
|  |  |  | 2 | 21/11/2023 |
|  | 38.422542652056 | 23.693027101994 | 1 | 08/11/2023 |
|  |  |  | 1 | 21/11/2023 |
| Laconia | 36.988031570859 | 22.437130051193 | 1 | 11/09/2023 |
|  | 37.001954006542 | 22.447172853449 | 14 | 11/09/2023 |
|  |  |  | 5 | 20/9/2023 |
| Lesbos | 39.323320512395 | 26.173744938518 | 6 | 24/10/2023 |
|  | 39.279835065579 | 26.013244550161 | 2 | 3/11/2023 |
|  |  |  | 2 | 15/11/2023 |
|  | 39.273650015936 | 26.013496368879 | 2 | 3/11/2023 |
|  |  |  | 2 | 15/11/2023 |
|  | 39.208609485532 | 26.222953104988 | 1 | 10/11/2023 |
|  | 39.21176330291 | 26.225287755258 | 2 | 10/11/2023 |
|  | 39.334286142227 | 26.192414969851 | 4 | 15/11/2023 |
| Heraklion | 35.328762376066 | 25.073086206472 | 4 | 21/09/2023 |
|  | 35.319743202134 | 25.054958383035 | 9 | 21/09/2023 |
| Lasithi | 35.189115043656 | 25.477961320662 | 8 | 15/09/2023 |
|  | 35.190569131932 | 25.485126579484 | 9 | 15/09/2023 |

**Suppl. Table S2** Primers used for the insecticide resistance assays.

| **IRAC mechanism** | **Gene**  **[GenBank Accession]** | **Assay** | **Type** | **Oligo name*** | **Sequence 5’→3’** | **Reference** |
| --- | --- | --- | --- | --- | --- | --- |
| **11** Microbial disruptors of insect midgut membranes *Bacillus thuringiensis* and the insecticidal proteins they produce | *ABCC2*  [KY489760] | R1: +GC insertion | qPCR | For_R1 | TGGAGGCCGAAGAGAGACA | ^1^ |
|  |  |  |  | Rev_R1 | AGGAGTTGACTGACTTCATGTACCT |  |
|  |  |  |  | Probe_wt_R1 | HEX-CCAAGCACATCCCACT-MGB |  |
|  |  |  |  | Probe_mut_R1 | FAM-CAAGCACATCGCCCACT-MGB |  |
|  |  | R2: A>G SNP | qPCR | For_R2 | AGGGTAACGTTCATTGTGATAATACC |  |
|  |  |  |  | Rev_R2 | CGCTGACTGGAAACATGATTAGTG |  |
|  |  |  |  | Probe_wt_R2 | *HEX*-CATCCAACAATACTTC-*MGB* |  |
|  |  |  |  | Probe_mut_R2 | *FAM*-CCATCCAACAGTACTTC-*MGB* |  |
|  |  | GY deletion P799K/R | PCR-Sanger seq. | **For_GYd_799** | **AGTGGGATGTGCTTGGGAG** | This study** |
|  |  |  |  | Rev_GYd_799 | AGTACTACGCTGCCGTGSA |  |
| **28** Ryanodine receptor modulators  Diamides | *RyR*  [MK226188] | I4790M/K | qPCR | For_4790 | AGGACGACGATGCACTAGAAG | ^2^ |
|  |  |  |  | Rev_4790 | AGACCRTCRAACTCCAATTTACG |  |
|  |  |  |  | Probe_wt_4790 | *HEX*-CTCGCTATACTCATCG-*MGB* |  |
|  |  |  |  | Probe_mut_4790M | *FAM*-TCGCTATGCTCATCG-*MGB* |  |
|  |  |  |  | Probe_mut_4790K | *FAM*-CTCGCTAAACTCATCGG-*MGB* |  |
|  |  | G4946E | PCR-Sanger seq. | **For_4946** | **GTGATGGGCAACTTCAAC** | This study*** |
|  |  |  |  | Rev_4946 | TTTTCCGTTATGCGTGAC |  |
| **5** nAChR allosteric modulators – Site I  Spinosyns | *nAChR*  [MW557608] | G275E | PCR-Sanger seq. | **For_275** | **TTCACCTTGCCTCCAGATTCT** | ^3^ |
|  |  |  |  | Rev_275 | AGTACAGGAACACGCATCGAAT |  |
|  |  | IIA deletion | PCR-Sanger seq. | **For_IIAd** | **TCTACAGAGCGAACTTGTTGCA** |  |
|  |  |  |  | Rev_IIAd | CTACGACACCATGATGTGCG |  |
| **3** Sodium channel modulators  **A** Pyrethroids, Pyrethrins | *Vgsc*  [MN966968] | M918T  T929I  L932F  I936V | PCR-Sanger seq. | **For_kdr1** | **GGTTCCGCAGTAGCATAGGTGA** |  |
|  |  |  |  | Rev_kdr1 | AGTTACTCCATGATCACTTTGCTTG |  |
|  |  | L1014F  F1020S | PCR-Sanger seq. | **For_kdr2** | **CAGATTACGTAGACCGTTTCCCG** |  |
|  |  |  |  | Rev_kdr2 | CGTCGGCTACGTTACGTTTCAC |  |
| **22** Voltage-dependent sodium channel blockers  **A** Oxadiazines | *Vgsc*  [MN966968] | F1845Y  V1848I | PCR-Sanger seq. | **For_kdr3** | **ATGGTATCATCAACGAGGAAGAGTG** |  |
|  |  |  |  | Rev_kdr3 | ATGTCGTAATCGTCGTCTGTGAG |  |
| **6** GluCl allosteric modulators  Avermectins | *GluCl*  [Gene ID: 118277865] | A308V  G314D | PCR-Sanger seq. | **For_glu** | **GTAACGACACTCCTCACGATGG** |  |
|  |  |  |  | Rev_glu | CGTGGTGTTGCTATCTGTGTCG |  |
| **1** AChE inhibitors  **A** Carbamates \| **B** Organophosphates | *Ace-1*  [KC435023] | A201S  G227A  F290V | PCR-Sanger seq. | **For_ace1** | **TCGGGTAATGCTGGTCTTTTTG** |  |
|  |  |  |  | Rev_ace1 | AAAGTAGTAACCTTCCTCCGTATTGG |  |

*The sequencing primer in each case is indicated with bold letters.

**The cycling conditions comprised of 95 ∘C for 3 min, followed by 40 cycles at 95 ∘C for 30 s, **52 ∘C** for 30 s and 72 ∘C for 1 min, and a final elongation step at 72 ∘C for 5 min.

*** The cycling conditions comprised of 95 ∘C for 5 min, followed by 40 cycles at 95 ∘C for 30 s, **56 ∘C** for 45 s and 72 ∘C for 45s, and a final elongation step at 72 ∘C for 10 min.

1. Flagel L, Lee YW, Wanjugi H, Swarup S, Brown A, Wang J, et al. Mutational disruption of the ABCC2 gene in fall armyworm, *Spodoptera frugiperda*, confers resistance to the Cry1Fa and Cry1A.105 insecticidal proteins. *Sci Rep*  **8**: 7255 (2018).

2. Okuma DM, Cuenca A, Nauen R and Omoto C. Large-Scale monitoring of the frequency of ryanodine receptor target-site mutations conferring diamide resistance in Brazilian field populations of fall armyworm, *Spodoptera frugiperda* (Lepidoptera: Noctuidae). *Insects*  **13** (2022).

3. Wang HH, Zhao R, Gao J, Zhang L, Zhang S, Liang P, et al. Genetic architecture and insecticide resistance in Chinese populations of *Spodoptera frugiperda*. *J Pest Sci*  **96**: 1595-1610 (2023).


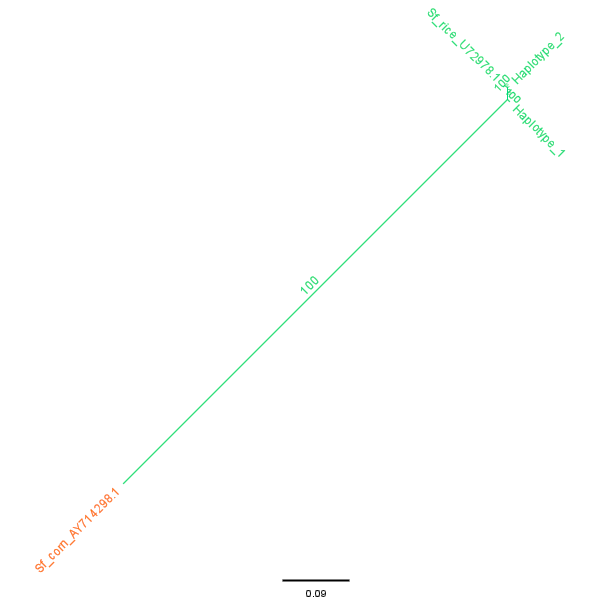


**Suppl. Figure S1 Strain ID analysis based on COI.** Using deposited sequences identified for these two strains (Sf_corn_AY714298.1 and Sf_rice_U72978.1), we conclude that the two haplotypes found in Greece correspond to the rice strain, for the COI gene region amplified by the primers LCO-1490 and HCO-2198. (Geneious Prime 2023.0.1 software, based on Neighbour joining methodology).

**
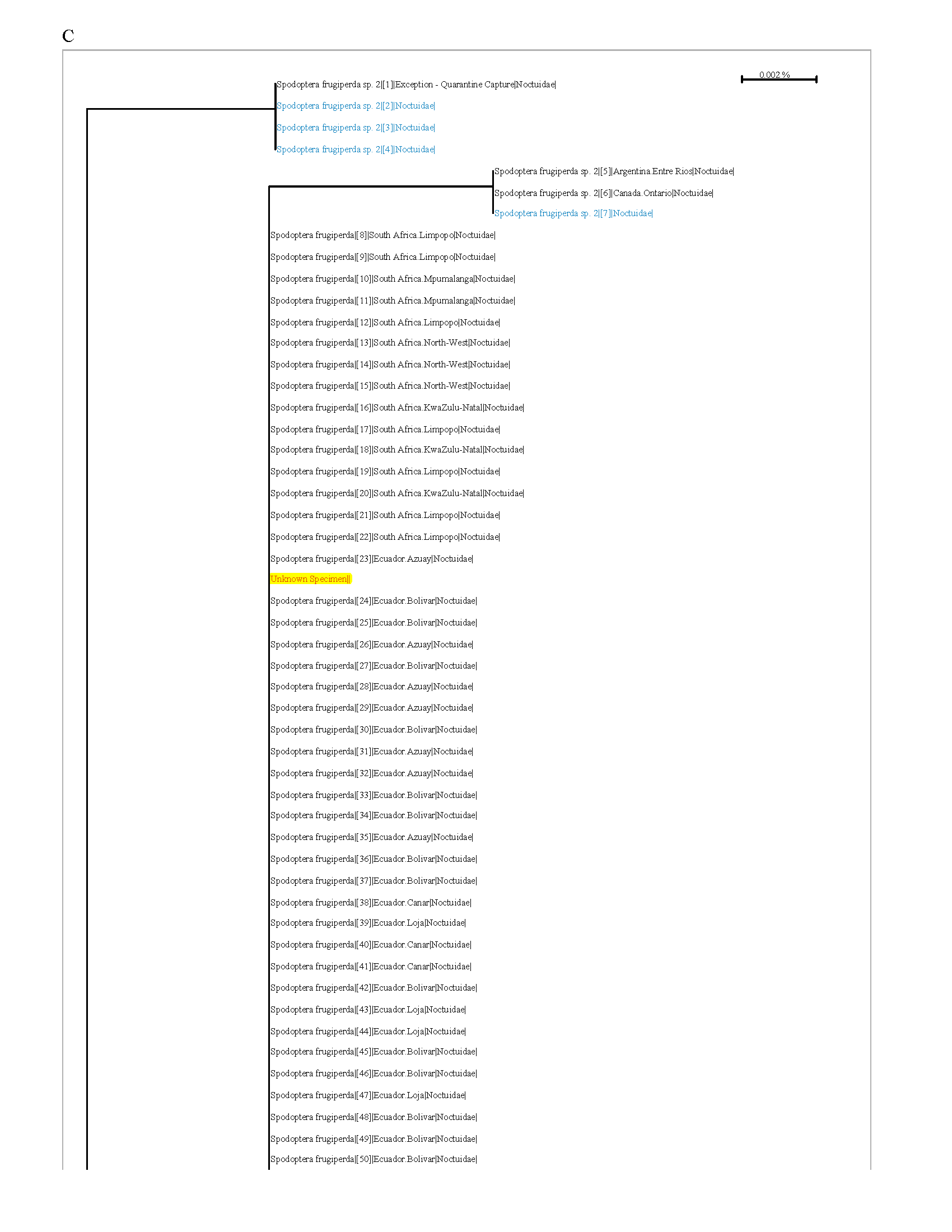
**

**
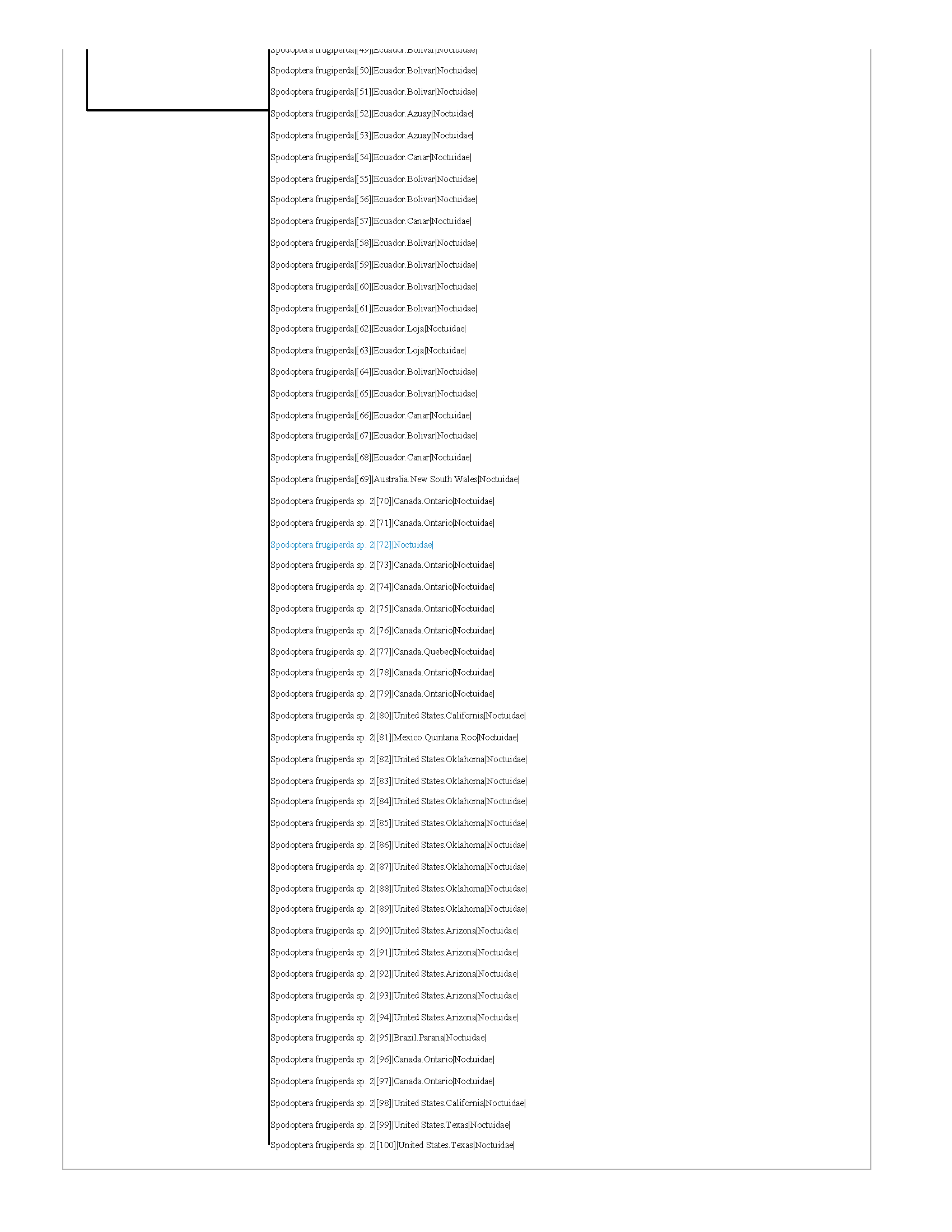
**

**Suppl. Figure S2 Haplotype 1 analysis.** The COI gene region of DNA (658bp) was compared with deposited sequences available in NCBI and BOLD databases to determine the origin of the insects. The origin was investigated by comparing the sequence of haplotype 1 (yellow highlight) with the top 100 deposited sequences from the BOLD database that had 100% matches (Neighbour joining pattern). Haplotype 1 is genetically closest, in descending order, to sequences from the following regions: South Africa, Ecuador, Australia, Canada, USA, Mexico, and Brazil.

**
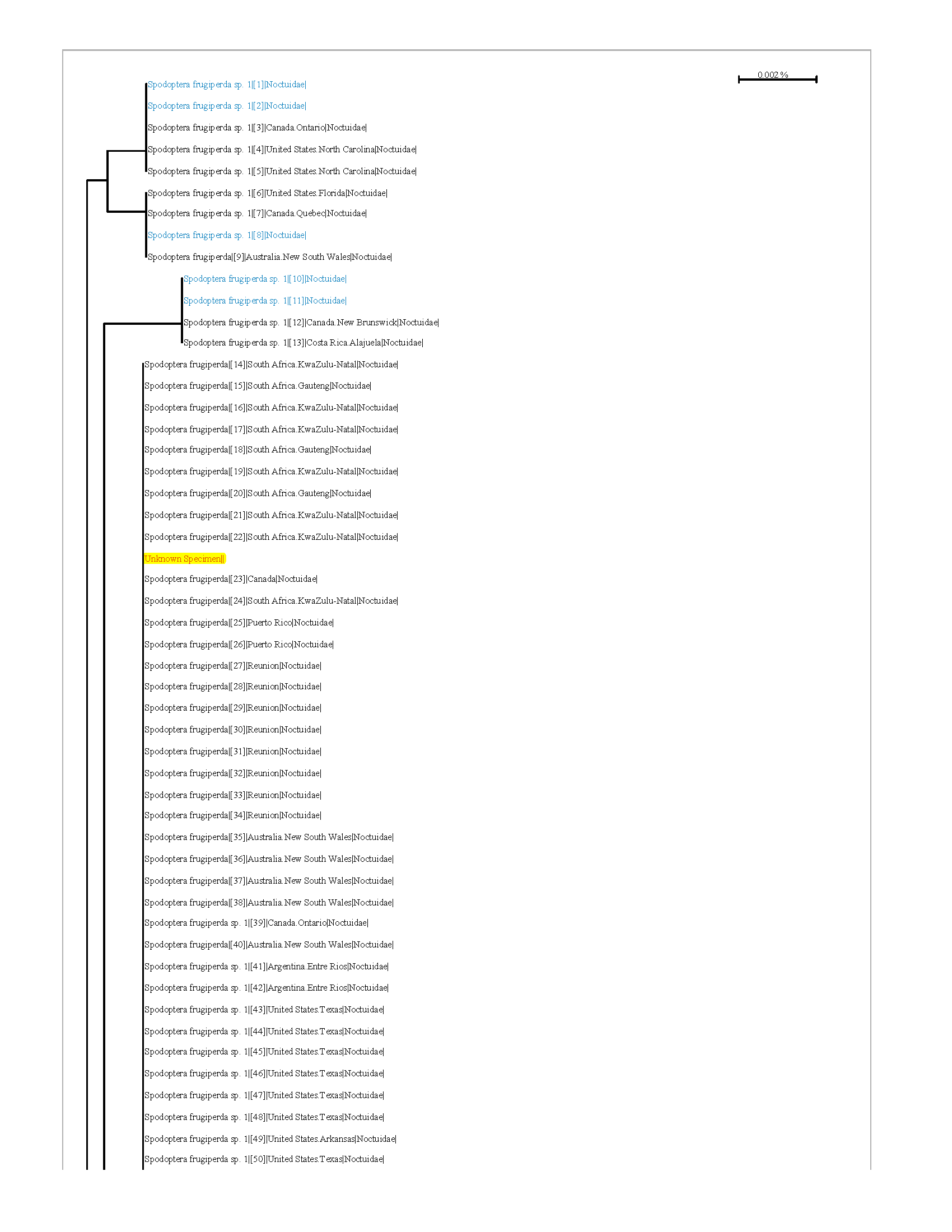
**

**
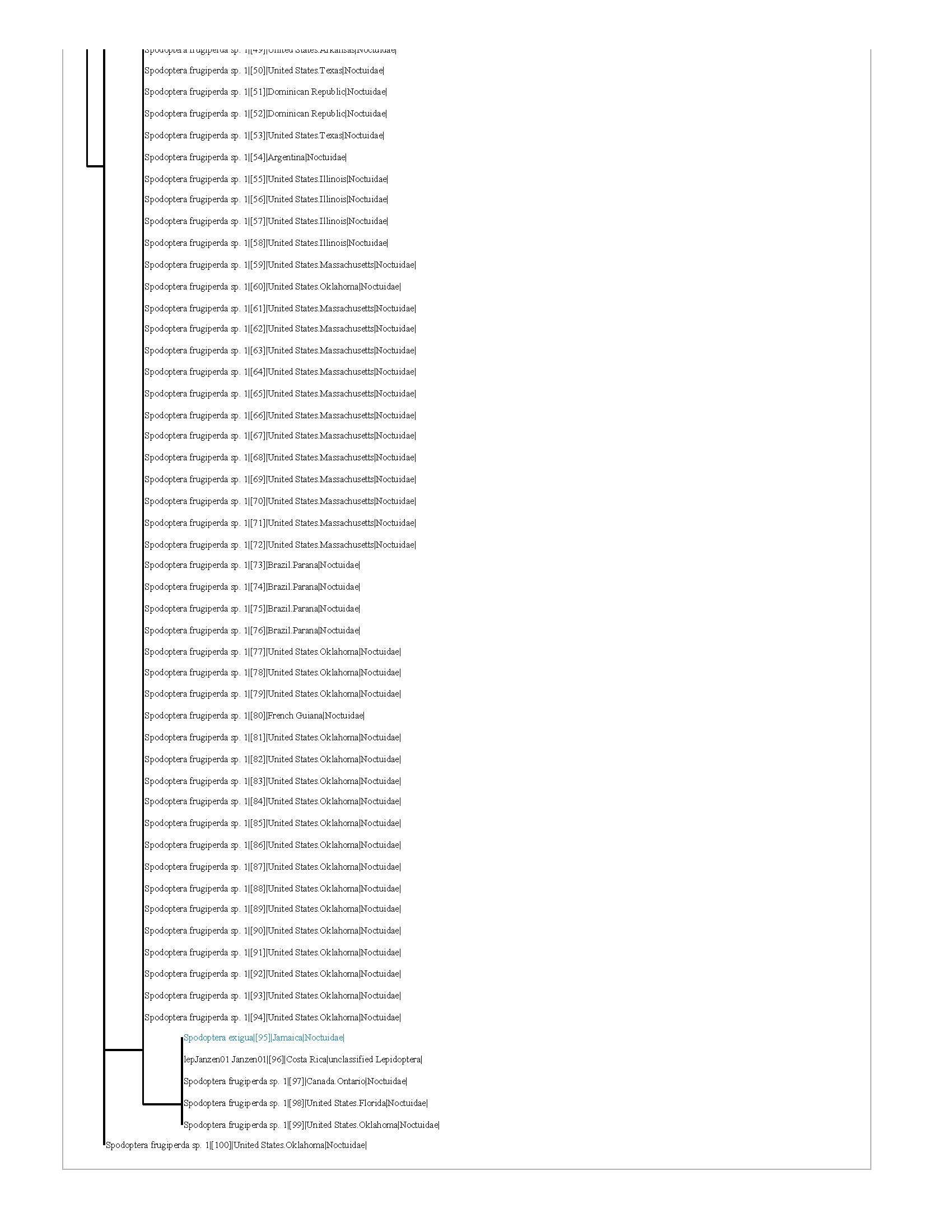
**

**Suppl. Figure S3 Haplotype 2 analysis.** The COI gene region of DNA (658bp) was compared with deposited sequences available in NCBI and BOLD databases to determine the origin of the insects. The origin was investigated by comparing the sequence of haplotype 2 (yellow highlight) with the top 100 deposited sequences from the BOLD database that had 100% matches (Neighbour joining pattern). Haplotype 2 is genetically closest to sequences from the following regions: South Africa, Canada, Puerto Rico, Réunion (east of Madagascar), Australia, Argentina, USA, Dominican Republic, Brazil, and French Guiana.

**Suppl. Table S3** Monitoring for mutations associated with diamide resistance (IRAC mechanism 28).

| **IRAC mechanism** | **28** Ryanodine receptor modulators  Diamides | |
| --- | --- | --- |
| **Gene**  **(mutation)** | ***RyR***  **(I4790M/K)** | ***RyR***  **(G4946E)** |
| **Population** | **Resistant mutation allelic frequencies**  (het/hom), N of alleles | |
| **East Attica** | **0.00%**  (0/0)  N = 40 | **0.00%**  (0/0)  N = 34 |
| **Euboea** | **0.00%**  (0/0)  N = 40 | **0.00%**  (0/0)  N = 34 |
| **Laconia** | **0.00%**  (0/0)  N = 40 | **0.00%**  (0/0)  N = 40 |
| **Lesbos** | **0.00%**  (0/0)  N = 42 | **0.00%**  (0/0)  N = 40 |
| **Heraklion** | **0.00%**  (0/0)  N = 26 | **0.00%**  (0/0)  N = 26 |
| **Lasithi** | **0.00%**  (0/0)  N = 34 | **0.00%**  (0/0)  N = 34 |

het = number of heterozygous individuals; hom = number of homozygous mutant individuals.

**Suppl. Table S4** Monitoring for mutations associated with **nicotinic acetylcholine receptor (nAChR) allosteric modulators – Site I** (IRAC mechanism 5).

| **IRAC mechanism** | **5** Nicotinic acetylcholine receptor (nAChR) allosteric modulators – Site I  Spinosyns | |
| --- | --- | --- |
| **Gene**  **(mutation)** | ***nAChR***  **(G275E)** | ***nAChR***  **(IIA deletion)** |
| **Population** | **Resistant mutation allelic frequencies**  (het/hom), N of alleles | |
| **East Attica** | **0.00%**  (0/0)  N = 32 | **0.00%**  (0/0)  N = 32 |
| **Euboea** | **0.00%**  (0/0)  N = 30 | **0.00%**  (0/0)  N = 28 |
| **Laconia** | **0.00%**  (0/0)  N = 24 | **0.00%**  (0/0)  N = 26 |
| **Lesbos** | **0.00%**  (0/0)  N = 28 | **0.00%**  (0/0)  N = 32 |
| **Heraklion** | **0.00%**  (0/0)  N = 18 | **0.00%**  (0/0)  N = 18 |
| **Lasithi** | **0.00%**  (0/0)  N = 22 | **0.00%**  (0/0)  N = 26 |

het = number of heterozygous individuals; hom = number of homozygous mutant individuals.

**Suppl. Table S5** Monitoring for mutations associated with pyrethroid resistance (IRAC mechanism 3 A).

| **IRAC mechanism** | **3** Sodium channel modulators  **A** Pyrethroids, Pyrethrins | | | | | |
| --- | --- | --- | --- | --- | --- | --- |
| **Gene**  **(mutation)** | ***vgsc***  **(M918T)** | ***vgsc***  **(T929I)** | ***vgsc***  **(L932F)** | ***vgsc***  **(I936V)** | ***vgsc***  **(L1014F)** | ***vgsc***  **(F1020S)** |
| **Population** | **Resistant mutation allelic frequencies**  (het/hom), N of alleles | | | | | |
| **East Attica** | **0.00%**  (0/0)  N = 34 | **0.00%**  (0/0)  N = 32 | **0.00%**  (0/0)  N = 32 | **0.00%**  (0/0)  N = 32 | **0.00%**  (0/0)  N = 36 | **0.00%**  (0/0)  N = 36 |
| **Euboea** | **0.00%**  (0/0)  N = 38 | **0.00%**  (0/0)  N = 38 | **0.00%**  (0/0)  N = 38 | **0.00%**  (0/0)  N = 38 | **0.00%**  (0/0)  N = 38 | **0.00%**  (0/0)  N = 38 |
| **Laconia** | **0.00%**  (0/0)  N = 34 | **0.00%**  (0/0)  N = 34 | **0.00%**  (0/0)  N = 34 | **0.00%**  (0/0)  N = 34 | **0.00%**  (0/0)  N = 40 | **0.00%**  (0/0)  N = 40 |
| **Lesbos** | **0.00%**  (0/0)  N = 30 | **0.00%**  (0/0)  N = 30 | **0.00%**  (0/0)  N = 30 | **0.00%**  (0/0)  N = 30 | **0.00%**  (0/0)  N = 38 | **0.00%**  (0/0)  N = 38 |
| **Heraklion** | **0.00%**  (0/0)  N = 14 | **0.00%**  (0/0)  N = 14 | **0.00%**  (0/0)  N = 14 | **0.00%**  (0/0)  N = 14 | **0.00%**  (0/0)  N = 26 | **0.00%**  (0/0)  N = 26 |
| **Lasithi** | **0.00%**  (0/0)  N = 16 | **0.00%**  (0/0)  N = 16 | **0.00%**  (0/0)  N = 16 | **0.00%**  (0/0)  N = 16 | **0.00%**  (0/0)  N = 32 | **0.00%**  (0/0)  N = 32 |

het = number of heterozygous individuals; hom = number of homozygous mutant individuals.

**Suppl. Table S6** Monitoring for mutations associated with oxadiazine resistance (IRAC mechanism 22 A).

| **IRAC mechanism** | **22** Voltage-dependent sodium channel blockers  **A** Oxadiazines | |
| --- | --- | --- |
| **Gene (mutation)** | ***vgsc***  **(F1845Y)** | ***vgsc***  **(V1848I)** |
| **Population** | **Resistant mutation allelic frequencies**  (het/hom), N of alleles | |
| **East Attica** | **0.00%**  (0/0)  N = 38 | **0.00%**  (0/0)  N = 38 |
| **Euboea** | **0.00%**  (0/0)  N = 40 | **0.00%**  (0/0)  N = 40 |
| **Laconia** | **0.00%**  (0/0)  N = 34 | **0.00%**  (0/0)  N = 34 |
| **Lesbos** | **0.00%**  (0/0)  N = 38 | **0.00%**  (0/0)  N = 38 |
| **Heraklion** | **0.00%**  (0/0)  N = 24 | **0.00%**  (0/0)  N = 24 |
| **Lasithi** | **0.00%**  (0/0)  N = 32 | **0.00%**  (0/0)  N = 32 |

het = number of heterozygous individuals; hom = number of homozygous mutant individuals.

**Suppl. Table S7** Monitoring for mutations associated with avermectin resistance (IRAC mechanism 6).

| **IRAC mechanism** | **6** Glutamate-gated chloride channel (GluCl) allosteric modulators  Avermectins | |
| --- | --- | --- |
| **Gene (mutation)** | ***GluCl***  **(A308V)** | ***GluCl***  **(G314D)** |
| **Population** | **Resistant mutation allelic frequencies**  (het/hom), N of alleles | |
| **East Attica** | **0.00%**  (0/0)  N = 40 | **0.00%**  (0/0)  N = 40 |
| **Euboea** | **0.00%**  (0/0)  N = 40 | **0.00%**  (0/0)  N = 40 |
| **Laconia** | **0.00%**  (0/0)  N = 38 | **0.00%**  (0/0)  N = 38 |
| **Lesbos** | **0.00%**  (0/0)  N = 40 | **0.00%**  (0/0)  N = 40 |
| **Heraklion** | **0.00%**  (0/0)  N = 26 | **0.00%**  (0/0)  N = 26 |
| **Lasithi** | **0.00%**  (0/0)  N = 34 | **0.00%**  (0/0)  N = 34 |

het = number of heterozygous individuals; hom = number of homozygous mutant individuals.
